# A method making fewer assumptions gave the most reliable estimates of exposure–outcome associations in stratified case–cohort studies

**DOI:** 10.1016/j.jclinepi.2015.04.007

**Published:** 2015-12

**Authors:** Edmund Jones, Michael J. Sweeting, Stephen J. Sharp, Simon G. Thompson, EPIC-InterAct Consortium

**Affiliations:** a Cardiovascular Epidemiology Unit, Department of Public Health and Primary Care, University of Cambridge, Strangeways Research Laboratory, Worts' Causeway, Cambridge, CB1 8RN, UK; b Medical Research Council Epidemiology Unit, University of Cambridge School of Clinical Medicine, Box 285, Institute of Metabolic Science, Cambridge Biomedical Campus, Cambridge, CB2 0QQ, UK

**Keywords:** Case–cohort study, Cox model, Hazard ratio, Meta-analysis, Stratification, Subcohort selection

## Abstract

**Objective:**

A case–cohort study is an efficient epidemiological study design for estimating exposure–outcome associations. When sampling of the subcohort is stratified, several methods of analysis are possible, but it is unclear how they compare. Our objective was to compare five analysis methods using Cox regression for this type of data, ranging from a crude model that ignores the stratification to a flexible one that allows nonproportional hazards and varying covariate effects across the strata.

**Study Design and Setting:**

We applied the five methods to estimate the association between physical activity and incident type 2 diabetes using data from a stratified case–cohort study and also used artificial data sets to exemplify circumstances in which they can give different results.

**Results:**

In the diabetes study, all methods except the method that ignores the stratification gave similar results for the hazard ratio associated with physical activity. In the artificial data sets, the more flexible methods were shown to be necessary when certain assumptions of the simpler models failed. The most flexible method gave reliable results for all the artificial data sets.

**Conclusion:**

The most flexible method is computationally straightforward, and appropriate whether or not key assumptions made by the simpler models are valid.

**What is new?**

**Key findings**
• This article compares five ways of analyzing stratified case–cohort studies, using data from the EPIC-InterAct study and artificial data sets. A two-stage Cox model with random-effect meta-analysis gave the most reliable results in the widest variety of scenarios and is a flexible model that makes fewer assumptions than the other models investigated.
• Three different estimators to account for the stratified case–cohort design were investigated in combination with the proposed models. All estimators performed poorly when the stratification was not incorporated in the model.

**What this adds to what was known**
• This article provides a detailed comparison and discussion of different analysis methods for stratified case–cohort studies, which is lacking in the existing literature. It presents the assumptions, together with the advantages and disadvantages of each proposed model, and makes recommendations for epidemiologists and applied statisticians who want to analyze data from studies with this design.

**What is the implication and what should change now?**
• A flexible two-stage Cox model with random-effect meta-analysis should be routinely considered for analyzing stratified case–cohort studies where the strata may be confounding the exposure–outcome relationship. Combined with a Prentice estimator, this approach provides reliable results and is generally recommended. An exception may be when there are very few events or strata that may lead to difficulties in fitting the two-stage random-effect model.

## 1 Introduction

A case–cohort study is nested within a prospective cohort study and is an efficient design because full covariate data are only gathered on the cases (participants who have an event during the follow-up period) and the subcohort. The subcohort is a randomly selected subset of the full cohort at baseline and therefore includes some future incident cases. The proportion of the cohort selected to be in the subcohort is called the sampling fraction. Obtaining data on the full cohort can be expensive, for example if biomarkers or DNA from blood samples are required, so the case–cohort design is more cost-effective than the full cohort study, especially when the event of interest is rare. Another advantage of the case–cohort design is that the same subcohort can be used to study multiple disease outcomes, though if exposures corresponding to different case definitions are measured at different times then batch effects might be a problem.

The selection of the subcohort is sometimes stratified on one or more covariates that are available in all cohort members, to improve efficiency or achieve a similar distribution of these covariates between the cases and the subcohort. The cohort is divided into strata, each with a potentially different sampling fraction for selecting participants to be in the subcohort. For data from this type of study, Borgan et al. [1] described how to fit a Cox proportional hazards model, for estimating exposure–outcome associations, in a way that aims to account for the stratified design. Recent articles [2], [3] mentioned only this method, but there are several other possibilities. In this article we describe five models, apply them to a case–cohort study, compare their performance using artificial data, and discuss their advantages and disadvantages.

We first describe approaches used to fit a Cox model to case–cohort data from an unstratified study, since these form the basis of the five methods for stratified studies, which we describe next. We apply the methods for stratified studies to data from the EPIC-InterAct study [4] (hereafter known as InterAct), which is a stratified case–cohort study of incident type 2 diabetes in 26 centers across Europe. We then use artificial data sets to show how the five methods can give different results. Finally, we discuss these findings and make recommendations for researchers analyzing data from stratified case–cohort studies.

## 2 Methods for unstratified case–cohort studies

We first review the use of Cox regression models [5] to analyze data from an unstratified case–cohort study, where the subcohort has been selected by simple random sampling (each participant has equal probability of being in the subcohort). If participant *i* has exposure of interest $x_{i}$ with associated log hazard ratio *β,* and confounder $z_{i}$ with log hazard ratio *γ*, then the model for the hazard function is

$$h_{i}\left(t\right)=h_{0}\left(t\right)\;\exp\left(\beta x_{i}+\gamma z_{i}\right)\text{.}$$

The baseline hazard $h_{0}\left(t\right)$ is common to all participants. If there is more than one confounder, then *γ* and $z_{i}$ are vectors and $\gamma z_{i}$ is replaced by $\gamma^{T}z_{i}$.

In a full cohort study, the partial likelihood function would be maximized to estimate *β* [5]. In a case–cohort study, this is not possible, because $x_{i}$ and $z_{i}$ are only known for the cases and members of the subcohort. The partial likelihood function is therefore approximated by a pseudolikelihood, of which several versions have been proposed that correspond to slightly different estimators. Simulations have shown that the estimator of Prentice [6] leads to estimates of *β* and its standard error that most closely resemble those that would have been obtained from the corresponding full cohort study [7]. Prentice's estimator also has desirable asymptotic properties [8]. One alternative is the estimator of Barlow [9], which, unlike Prentice's, directly involves the sampling fraction and approximates the likelihood from the full cohort study. These two estimators are explained in detail in Appendix A, in the supplementary material at www.jclinepi.com.

Kim [10] has recently carried out a more thorough comparison of estimators for case–cohort data using simulated data sets. He found that inverse probability weighting estimators are the most powerful, but overall the differences between estimators are small when the proportion of cases in the full cohort is less than 10% and negligible when it is less than 1%. The differences also decrease as the size of the data set (the number of observations) increases.

For case–cohort data, the asymptotic theory for variances in the standard Cox model does not hold [6], and it is necessary to use “robust” variance estimates [9], [11], [12]. The assumption of proportional hazards can be checked by adding to the model an interaction between a baseline covariate and the underlying timescale, or by using an adaptation of Schoenfeld residuals [13].

## 3 Methods for stratified case–cohort studies

We now describe methods for analyzing data from a stratified case–cohort study. Each stratum has its own sampling fraction, and within a stratum each participant has equal probability of being selected to be in the subcohort. For ease of exposition we will refer to the strata as centers, since this is a common form of stratification in practice, but the strata can also be defined in terms of one or more other covariates, for example a correlate of the exposure (see the last paragraph of the discussion).

Suppose that participant *i* in center *j* has exposure $x_{ij}$ and confounder(s) $z_{ij}$, and the log hazard ratios corresponding to the exposure and confounder(s) are *β* and *γ* respectively. We describe five Cox regression models of increasing complexity for exposure–outcome associations in a stratified case–cohort study and methods for estimating their parameters.

### *Model I*

Single Cox model, with center not included in the model:

$$h_{ij}\left(t\right)=h_{0}\left(t\right)\;\exp\left(\beta x_{ij}+\gamma z_{ij}\right)\text{.}$$

The parameters of this model can be estimated using the estimators of (a) Prentice, (b) Barlow, or (c) Borgan (estimator III in [1]). Estimator (c) is specially intended for the purpose of fitting an unstratified Cox model to data from a stratified case–cohort study. Estimators (a) and (b) are not expected to be appropriate, whereas (c) is formally correct but computationally complex and has only recently become available in standard software. For (c), the usual robust variance estimator is not valid, so an asymptotic estimator is used instead [11].

### *Model II*

Single unstratified Cox model, including center as a categorical covariate:

$$h_{ij}\left(t\right)=h_{0}\left(t\right)\;\exp\left(\beta x_{ij}+\gamma z_{ij}+\delta_{j}\right)\text{.}$$

This model assumes proportional hazards between the different centers, with $\delta_{j}$ representing the log hazard ratio for center *j* relative to center 1 (so that $\delta_{1}=0$). The three estimators used for Model I can also be used for Model II.

### *Model III*

Single Cox model with the baseline hazard stratified by center:

$$h_{ij}\left(t\right)=h_{0j}\left(t\right)\;\exp\left(\beta x_{ij}+\gamma z_{ij}\right)\text{.}$$

This model does not assume proportional hazards between the centers. Instead it gives each center *j* its own baseline hazard $h_{0j}\left(t\right)$. Prentice's and Barlow's estimators can be used. The pseudolikelihood for Barlow's estimator is similar to the pseudolikelihood for Borgan's estimator, so in this article we use Barlow's estimator with Models I and II to compare it with Borgan's.

### *Model IV*

Separate Cox model for each center, assuming a common log hazard ratio for the exposure in all centers:

$$h_{ij}\left(t\right)=h_{0j}\left(t\right)\;\exp\left(\beta x_{ij}+\gamma_{j}z_{ij}\right)\text{.}$$

This model allows the confounder effects to vary between centers, as represented by the parameters $\gamma_{j}$. The data can be analyzed by a two-stage method: first fit a separate Cox model to each center, using Prentice's estimator; then combine the estimates of *β* using fixed-effect meta-analysis. This model could also be fitted by a one-stage method, with a single pseudolikelihood for the whole data set, by including interaction terms between the center variables and the confounder(s).

### *Model V*

Separate Cox model for each center, with each center having its own log hazard ratio for the exposure, and these log hazard ratios following a normal distribution:

$$h_{ij}\left(t\right)=h_{0j}\left(t\right)\;\exp\left(\beta_{j}x_{ij}+\gamma_{j}z_{ij}\right)$$

$$\beta_{j}\;∼\;N\left(\beta ,\tau^{2}\right)\text{.}$$

Here $\tau^{2}$ is the between-study variance of the true center-specific log hazard ratios. This extends Model IV to allow the exposure–outcome association to vary between centers; β is now the average log hazard ratio. The model parameters can be estimated using the same two-stage approach as for Model IV but with random-effect (instead of fixed-effect) meta-analysis. In our analyses we used a moment estimator of $\tau^{2}$ [14]. Model V could also be fitted as a one-stage random-effects model, but this is computationally difficult for survival models and results have been shown to be very similar between one-stage and two-stage models [15].

The assumptions each model makes about the exposure effect, center effects, and confounder effect(s) are summarized in Table 1, and software functions for them are described in Appendix B.

## 4 Application of the models to InterAct

InterAct is a multicenter case–cohort study of incident type 2 diabetes nested within the EPIC-Europe cohort (EPIC is the European Prospective Investigation of Cancer and Nutrition) [4], [16]. The cohort from which the InterAct cases and subcohort members were sampled consisted of 340,234 participants, recruited from 1993 to 1999; this was smaller than the full EPIC-Europe cohort because blood samples were unavailable for some EPIC-Europe participants and because two of its countries did not participate in InterAct. The subcohort selection for InterAct was stratified by center, except in France, which was a single stratum (due to the small number of participants in each center in that country). We therefore regard France as a single center, so there are 21 centers in our analysis. In each center, the sampling fraction was chosen to be approximately 0.5–1% higher than the estimated baseline prevalence of type 2 diabetes in that center's population.

We estimate the association between self-reported physical activity and incident type 2 diabetes using data from InterAct and the five models previously described. This association has already been investigated and reported elsewhere [17]; here the same data are used to explore how the five different methods for stratified case–cohort data perform. Table 2 provides an overview of InterAct. Sampling fractions ranged from 2.6% to 10.5% across the centers. Physical activity was self-reported as 1 (inactive), 2 (moderately inactive), 3 (moderately active), or 4 (active) and included in the models as a continuous variable. The only confounder included was sex. In all models we used age in years as the timescale, as recommended for observational studies [18]. Borgan's estimator assumes there are no tied events, so tied event-times were changed by up to 0.01 days. Deaths from any cause were regarded as censored observations.

**Fig. 1:**
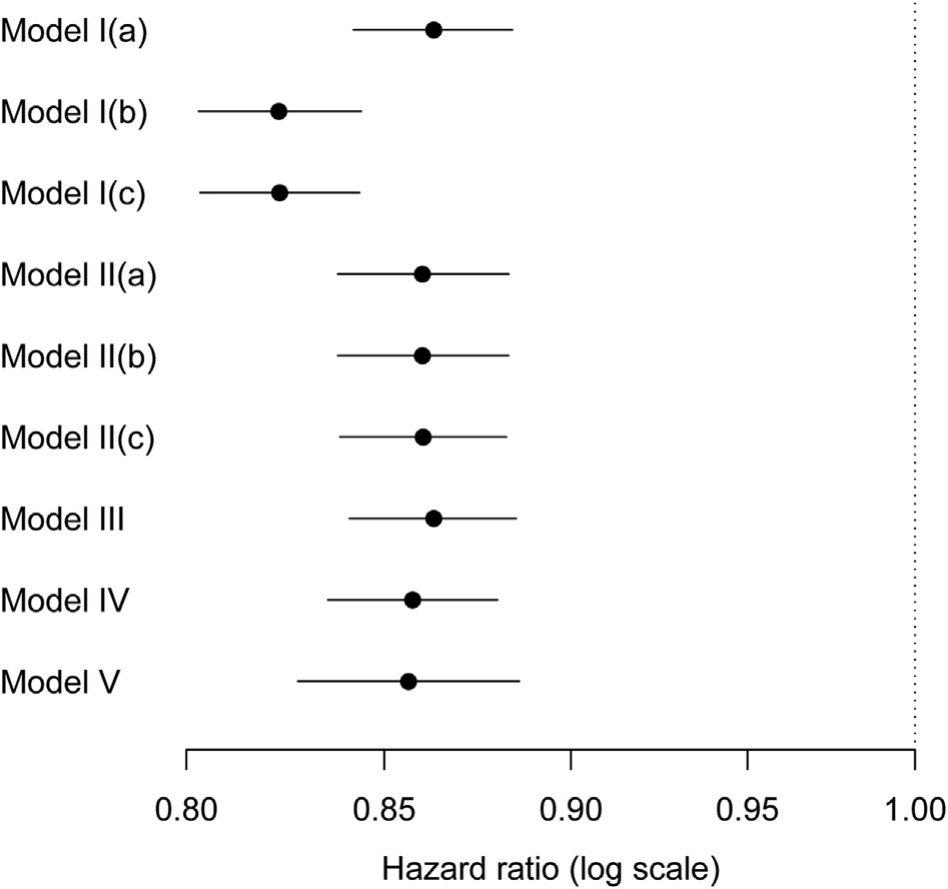
Estimates and 95% confidence intervals of the hazard ratio for diabetes per one-category increase in self-reported physical activity, using data from InterAct and Models I–V. Models I and II use the estimators of (a) Prentice, (b) Barlow, and (c) Borgan. Models III, IV, and V use only Prentice's estimator.

The results of applying Models I–V to the InterAct data are shown in Fig. 1. Greater physical activity is associated with a lower risk of diabetes. For most of the models and estimators the hazard ratios for physical activity are very similar. The only exceptions are the Barlow and Borgan estimators with Model I, which ignores center effects. The slightly lower estimates for Models IV and V indicate possible differential confounding by sex across centers. The wider confidence interval for Model V reflects the fact that there is heterogeneity in the hazard ratio across centers.

For the methods that use the Prentice estimator, the assumption of proportional hazards was tested by adding to the models an interaction between physical activity and the underlying timescale (age). In Models IV and V, meta-analyses of the interaction parameter estimates across centers were then performed [19]. The interaction terms were not statistically significant (*P* > 0.5 in all models). A test of proportional hazards between the 21 centers in Model II yielded $\chi_{20}^{2}$ = 159.0 (*P* < 0.001), suggesting that Models III–V should be preferable.

The published investigation of physical activity and incident type 2 diabetes [17] used Model V and found that the hazard ratio for a one-category increase in physical activity was 0.87 in men (with 95% confidence interval 0.80, 0.94) and 0.93 in women (0.89, 0.98). As well as analyzing the sexes separately, these analyses used more covariates and a different grouping of the centers, so the results are not directly comparable with those reported here.

## 5 Comparisons of the models using artificial data sets

Given the general similarity of the results for Models I–V found above, we next used artificial data sets to demonstrate circumstances in which the five models give different estimates of an exposure–outcome association. For each consecutive pair of models, we created an artificial data set from the more complex of the two models and then estimated the log hazard ratio using both models. A model was deemed to have estimated the hazard ratio accurately if the 95% confidence interval contained the true value. A set of four “realistic” data sets was created with specifications (size, sampling fractions, effect sizes, etc.) similar to the InterAct study. A set of four smaller “extreme” data sets was also created to show clearly the differences between the models.

We used a normally distributed exposure and a single binary-valued confounder. As above the strata for subcohort selection are referred to as “centers.” As far as possible, the same specifications were used for multiple data sets. The specifications are shown in Table 3 and computer code for generating the data is given in Appendix B. For Models I and II, we used only estimators (a) and (c), and for all data sets we also used Model V.

**Fig. 2:**
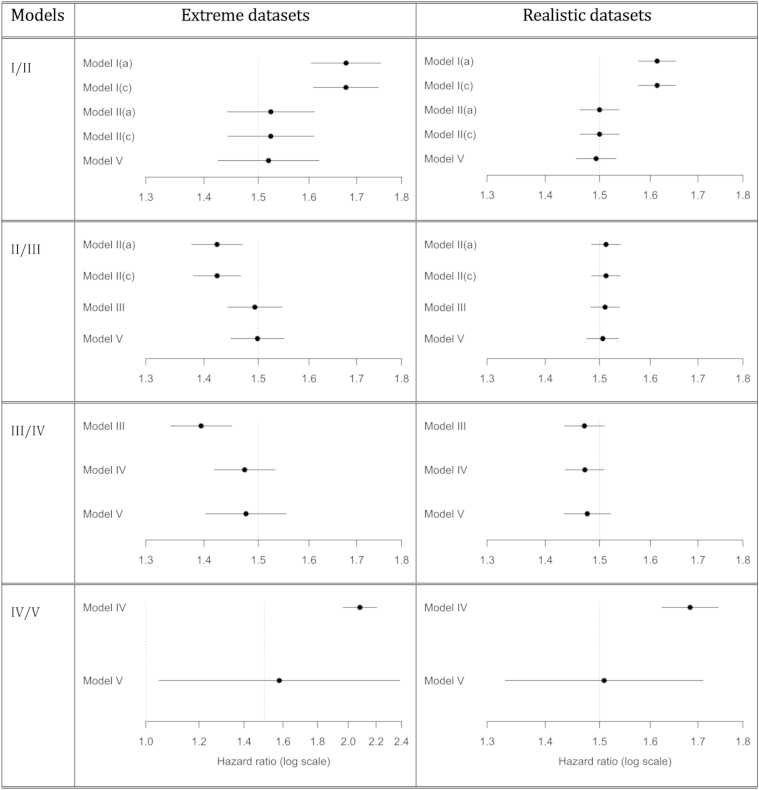
Estimates and 95% confidence intervals of the exposure hazard ratio for the artificial data sets. The true hazard ratio of 1.5 is marked by the dotted lines.

For all data sets the true value of the exposure hazard ratio was 1.5. The data sets for Models IV/V had different exposure hazard ratios in each center, but these were chosen so that the mean of the $\beta_{j}$’s (see section 3) was log1.5. Fig. 2 shows the estimated hazard ratios per unit increase in the exposure, and 95% confidence intervals, obtained from fitting the relevant pair of models to each data set. For the extreme data sets, the confidence intervals from the simpler models do not contain the true value of 1.5, whereas those from the more complex models do. For Models I/II and IV/V, the same is true with the realistic data sets. In all cases Model V appeared to provide a reliable analysis.

For the data sets for Models I/II, the centers with higher risks also had higher average exposure values. Hence center and exposure were confounded, and Model I's failure to allow for center effects led to incorrect results. For the data sets for Models II/III, nonproportional hazards across centers were achieved by using Weibull distributions with different shape parameters. Centers with higher risks also had higher average exposure values. Model II assumes proportional hazards across centers and so can give incorrect results as shown by the “extreme” example. For Models III/IV, the centers with higher risks and higher average exposure values also had lower hazard ratios for the confounder. Not allowing for the varying confounding effect across centers in Model III led to incorrect results with the “extreme” data set. For Models IV/V, larger centers had higher exposure hazard ratios. Larger centers are given more weight in fixed-effect than in random-effect meta-analysis, so Model IV gave higher hazard ratio estimates than Model V. The heterogeneity in the exposure hazard ratios also led to wide confidence intervals with Model V.

To investigate other aspects of the estimators' performance we performed a simulation study, simulating 200 data sets for each of the “realistic” specifications from Table 3 (a larger simulation study was impractical for computational reasons). For Models I and II we used only Prentice's estimator, because Borgan's estimator would be too time-consuming to calculate for this many data sets. As above, we fitted Model V to all the data sets, partly to see whether it was much less efficient than the simpler models. For each model we recorded the coverage rate, mean bias, and mean squared error of the exposure log hazard ratio, and for the more complex models we recorded the mean of the relative standard error compared to the simpler model—the larger this ratio, the less efficient the more complex model is. For 200 simulations, the standard error of the estimated coverage is approximately 0.015, which is small enough to show some of the differences between the models.

The results are shown in Table C.1 in Appendix C. According to the mean relative standard errors, the more complex models were mostly only slightly less efficient than the simpler ones, with the exception that Model V was much less efficient than Model IV when applied to the data sets for Models IV/V. However, Model V had less bias and thus much lower mean squared error than Model IV. Overall, in terms of coverage, mean bias, and mean squared error, the more complex models and Model V performed either very similarly to the simpler models or better than them, and Model V performed far better than Model IV.

## 6 Discussion

We have described five models for the analysis of data from a stratified case–cohort study, applied them to a study of physical activity and diabetes incidence, and used artificial data sets to investigate circumstances in which they can give different results. Model I, which took no account of the stratification, gave unreliable results in both the diabetes study and the artificial examples, regardless of which estimator was used. Even the Borgan estimator gave incorrect results with Model I in our examples. This suggests that the statistical model has to explicitly incorporate the stratification. It is also necessary to consider assumptions such as proportional hazards and homogeneous covariate effects across strata, as these may fail to hold in particular studies. However, we found that Model V, the most complex model, gave reliable results whether or not these assumptions held. Thus it is a method that can be generally recommended. All the models described are simple to fit with standard software (see Appendix B).

The main drawback of Model V is that if some strata have very few events, then fitting a separate Cox model for each one might not be possible. If that happens then data from an appropriate grouping of strata could be analyzed using Model II or III. Another drawback of Model V is that assessing the assumption of proportional hazards is cumbersome, since it needs to be done for each stratum and then an overall assessment made by meta-analysis of the nonproportionality parameter estimates [19]. Similarly, it is cumbersome to assess the shape of association when there is a separate model for each stratum. Another possible drawback is that random-effect meta-analysis is not appropriate if there are very few strata, because the estimate of the between-strata variance will not be precise.

There are several issues that we have not addressed. For example, we have not considered missing data or whether multiple imputation would be easier to perform in some models than others. If multiple imputation was used with Model V, it would need to be done in each stratum separately, using Rubin's rules, before the meta-analysis [20]. We have only considered methods based on Cox regression, with a nonparametric baseline hazard, since these seem to be used almost exclusively in practice [21], but parametric survival models for stratified case–cohort studies could be developed. These might have particular relevance for risk prediction [22], whereas the focus of this article has been on estimating the risk association of a particular exposure.

The artificial data sets were deliberately created to show that the models can give different results in certain plausible scenarios. Our main purpose was not to investigate the statistical properties of the five models, such as bias or coverage. However, the simulation study with 200 data sets for each of the realistic data set specifications gave similar results, providing reassurance about the stability of our findings. In particular, it provided evidence that Model V performs well in terms of bias and coverage and can be almost as efficient as the simpler models.

There are alternative possibilities for both the selection of the subcohort and the stratification in the analysis model. Subcohorts can be selected by more elaborate sampling schemes, such as using a formula in terms of baseline covariates to specify the relative probability of selection for each participant [23]. If the formula gives the same probability for different participants, for example because it uses only discrete-valued covariates, then this is similar to the stratified selection discussed in this article.

In terms of analysis, Model III can be adapted by stratifying the Cox model differently from the stratification of the subcohort-selection. For InterAct, the model could be stratified by country (a coarser stratification than the subcohort-selection), or by center and sex (a finer stratification than the subcohort-selection), or just by sex. Stratifying the model more finely than the subcohort-selection might be necessary if the assumption of proportional hazards was not met for some covariates. Similarly, Models IV and V could be adapted by using different subsets of the data for the separate Cox models.

In their discussion of different types of stratification, Langholz and Jiao [11] describe two situations for stratified case–cohort studies. In the “exposure stratified” situation, the subcohort selection is usually stratified according to a correlate of the exposure of interest, to improve the efficiency when estimating the exposure's effect size [1], and the data are analyzed with an unstratified Cox model. In the “confounder stratified” situation, the strata might be population groups or centers, and the data are analyzed with a stratified Cox model that uses the same stratification, to account for differences between the strata that might otherwise lead to bias. Our five models can be used regardless of the motivation behind the study design (although Model I should be avoided if the stratification variable is confounding the exposure–outcome association). However, we have focused more on stratifications that correspond to population groups or centers, as in InterAct and EPIC-CVD [24], which is another case–cohort study based on the EPIC-Europe cohort.

## Figures and Tables

**Table 1:** Summary of assumptions about the effects of exposure, centers, and confounder(s), in five models for data from stratified case–cohort studies

Model	Exposure effect	Center effects	Confounder effect(s)
I	Same in all centers	Not included in the model	Same in all centers
II	Same in all centers	Proportional effect in each center	Same in all centers
III	Same in all centers	Each center has its own baseline hazard	Same in all centers
IV	Same in all centers	Each center has its own baseline hazard	Varies by center
V	Varies by center	Each center has its own baseline hazard	Varies by center

Proportional hazards are assumed for all exposure and confounder effects.

**Table 2:** Summary statistics for the centers in the InterAct study

Center	Sampling fraction (%)	Size of case–cohort set^a^	Size of subcohort	Number of cases	Percent with high PA (subcohort)^b^	Percent male (subcohort)
France	2.86	867	588	288	44.0	0.00
Italy						
Florence	4.07	931	544	400	35.8	23.35
Varese	3.12	601	364	246	30.8	20.60
Ragusa	5.72	597	341	271	32.3	46.63
Turin	5.38	858	548	327	44.0	57.30
Naples	4.41	406	222	193	10.8	0.00
Spain						
Asturias	9.69	1,287	791	557	28.6	38.81
Granada	8.43	815	529	318	14.6	20.60
Murcia	10.48	1,264	761	549	25.5	31.80
Navarra	10.42	1,286	776	581	36.1	47.42
San Sebastian	9.54	1,237	731	559	36.4	46.65
UK						
Cambridge	4.44	1,747	989	787	38.3	42.77
Oxford	2.59	577	340	238	45.3	27.06
Netherlands						
Bilthoven	3.08	879	579	316	69.8	48.53
Utrecht	5.63	1,411	924	512	64.7	0.00
Germany						
Heidelberg	3.93	1,618	870	780	55.9	44.71
Potsdam	4.82	1,960	1,184	804	40.8	39.53
Sweden						
Malmo	7.09	3,556	1,929	1,757	39.7	40.38
Umea	4.08	1,845	1,015	865	47.7	48.87
Denmark						
Aarhus	3.91	1,265	665	640	57.1	52.48
Copenhagen	3.83	2,772	1,464	1,415	61.1	54.10
Total/overall	4.92	27,779	16,154	12,403	43.4	37.83

a The size of the case–cohort set is not equal to the size of the subcohort plus the number of cases, because the subcohort contains both cases and non-cases.

b Percentage of participants with physical activity (PA) = 3 or 4, meaning “moderately active” or “active.”

**Table 3:** Specifications for the artificial data sets

*Abbreviations*: Wb(a,b) is the Weibull distribution with scale *a* and shape *b*, which has hazard function $h\left(t\right)=\left(b/a\right){\left(t/a\right)}^{b-1}$; C*x* is “center *x*”; n/a is “not applicable.”

## Supplementary data

Appendices A–C
