# Supplementary material for: A method making fewer assumptions gave the most reliable estimates of exposure–outcome associations in stratified case–cohort studies
Source: J Clin Epidemiol. 2015 Dec;68(12):1397–405. doi: 10.1016/j.jclinepi.2015.04.007 (PMC4669309; doi:10.1016/j.jclinepi.2015.04.007)
Supplement: Appendices A–C [file mmc1.pdf]

Supplementary material for "A method making fewer assumptions gave the most reliable estimates of exposure–outcome associations in stratified case–cohort studies" (E. Jones et al., *Journal of Clinical Epidemiology*, 2015)

Appendix A: Pseudolikelihoods for data from unstratified case–cohort studies

This is an explanation of several estimators for unstratified case–cohort data. If participant i has covariates x_i then the hazard function is

$$h_i(t) = h_0(t)\exp(\beta^T x_i).$$

For the standard Cox model, as used for cohort studies, a participant i who has an event at time t_j contributes the following factor to the partial likelihood:

$$\frac{e^{\beta^T x_i}}{\sum_{k\in R(t_j)} e^{\beta^T x_k}},$$

where $R(t_j)$ is the set of participants who are at risk at time t_j. For case–cohort studies this has to be adapted, because x_k is only known for the participants in the case–cohort set. The partial likelihood is replaced by an approximation to it called the pseudolikelihood [1], and the contribution is often written as

$$\frac{e^{\beta^T x_i}}{Y_i(t_j)w_i(t_j)e^{\beta^T x_i} + \sum_{k\in S, k\neq i} Y_k(t_j)w_k(t_j)e^{\beta^T x_k}}$$

[2]. Here $Y_i(t_j)$ indicates whether participant i was at risk at time t_j, $w_i(t_j)$ is the weight assigned to participant i at time t_j, and S is the subcohort set. Two choices for the weights are shown in Table A.1.

Prentice's estimator is discussed in section 3 of the main text. In Barlow's estimator, subcohort non-cases and subcohort cases before their events are weighted by $1/\alpha$, where α is the sampling fraction, to approximate the contributions that would have occurred in the corresponding cohort study, so that the expected value of the denominator is its value in the cohort study. Prentice's and Barlow's estimators are sometimes listed alongside the estimator of Self & Prentice [3] as a set of three, but Self & Prentice's estimator was only proposed in order to prove asymptotic results about Prentice's. Many other estimators have been proposed [4].

Table A.1. Weights $w_i(t_j)$ in the pseudolikelihood, for the estimators of Prentice and Barlow. α is the sampling fraction.

	**Prentice [1]**	**Barlow [5]**
Non-subcohort case before event	0	0
Non-subcohort case at time of event	1	1
Subcohort case before event	1	$1/\alpha$
Subcohort case at time of event	1	1
Subcohort non-case	1	$1/\alpha$

Appendix B: Software for Models I to V and generating from the Cox model

For Models I(a), I(b), II(a), II(b), and III, we used Stata functions that are available at http://www.phpc.cam.ac.uk/ceu/research/erfc/stata/. To use these, install them as described on the webpage and then type "help stsetcco". For Methods IV and V we additionally used statsby and metan.

For Methods I(c) and II(c) we used an early version of a function from the R package "cchs" [6]. This package has since been publicly released and is available at http://cran.r-project.org/web/packages/cchs/ .

The R function cch, in the package "survival" [7][8], can fit Models I(a) and II(a) but not Model III.

To create the artificial datasets it was necessary to generate values from the Cox proportional hazards model. The following function, generateFromCoxPH, takes three arguments: the covariates x, the coefficients β, and the distribution that corresponds to the baseline hazard function, which is completely arbitrary. It returns a value from the Cox proportional hazards model, in other words a time to event. The third argument has to be supplied in the form of the distribution's inverse cumulative hazard function. The two lines in the body of generateFromCoxPH correspond to equation 6 in [9].

```
# Function to generate from the Cox proportional hazards model:
generateFromCoxPH <- function(x, b, inverseBaselineCumulativeHazard) {
      linearPredictor <- drop(crossprod(b,x))  # this is "bTx"
      inverseBaselineCumulativeHazard( rexp(1) * exp(-linearPredictor) )
}

# Three examples of functions to create inverse cumulative hazard functions
# that can then be passed to generateFromCoxPH (the second and third are for
# the Weibull distribution with two different parameterizations):
exponentialInverseCumulativeHazard <- function(lambda) {
      function(x) { x / lambda }
}
weibullInverseCumulativeHazard_A <- function(scale, shape) {
      function(x) { scale * x^(1/shape) }  # h(x)=(shape/scale)*(x/scale)^(shape-1)
}
weibullInverseCumulativeHazard_B <- function(scale, shape) {
      function(x) { (x/scale)^(1/shape) }  # h(x)=scale*shape*x^(shape-1)
}

# Example of how to use generateFromCoxPH:
generateFromCoxPH(x=c(0.73,1), b=log(c(1.5,0.8)), weibullInverseCumulativeHazard_A(30,4))
```

Appendix C: Results of the simulation study

Table C.1. Performance measures from the application of the models to 200 randomly generated datasets for each of the "realistic" specifications from Table 3 (in the main text).

Models datasets are intended to compare	Model	Coverage of nominal 95% confidence interval	Mean bias	Mean squared error	Mean relative SE compared to simple model
I/II	I(a)	0.000	0.0756	0.005823	–
	II(a)	0.970	0.0007	0.000128	1.040
	V	0.972	−0.0010	0.000124	1.078
II/III	II(a)	0.940	0.0009	0.000091	–
	III	0.935	−0.0002	0.000090	0.996
	V	0.950	−0.0010	0.000091	1.049
III/IV	III	0.946	0.0000	0.000133	–
	IV	0.950	−0.0018	0.000137	0.994
	V	0.960	−0.0014	0.000136	1.051
IV/V	IV	0.000	0.1133	0.013417	–
	V	1.000	0.0203	0.000929	3.089

References

[1] Prentice RL. A case–cohort design for epidemiological cohort studies and disease prevention trials. *Biometrika* 1986;73(1):1–11.

[2] Barlow WE, Ichikawa L, Rosner D, Izumi S. Analysis of case–cohort designs. *J Clin Epidemiol* 1999;52(12):1165–1172.

[3] Self SG, Prentice RL. Asymptotic distribution theory and efficiency results for case–cohort studies. *Ann Stat* 1988;16(1):64–81.

[4] Kulich M, Lin DY. Improving the efficiency of relative-risk estimation in case–cohort studies. *J Am Stat Assoc* 2004;99(467):832–844.

[5] Barlow WE. Robust variance estimation for the case–cohort design. *Biometrics* 1994;50(4):1064–72.

[6] Jones E. cchs: Cox model for case–cohort data with stratified subcohort-selection. R package version 0.1.0. Available from http://cran.r-project.org/web/packages/cchs/. Updated January 29, 2015. Accessed February 12, 2015.

[7] Therneau TM. A package for survival analysis in S. R package version 2.37-7. Available from http://cran.r-project.org/web/packages/survival/. Updated January 22, 2014. Accessed February 12, 2015.

[8] Therneau TM, Grambsch PM. *Modeling Survival Data: Extending the Cox Model.* New York, NY: Springer; 2000.

[9] Bender R, Augustin T, Blettner M. Generating survival times to simulate Cox proportional hazards models. *Stat Med* 2005;24(11):1713–1723.
